# Cervical Infantile Fibrosarcoma: a rare cause of paediatric parapharyngeal neck mass

**DOI:** 10.4322/acr.2020.189

**Published:** 2020-11-20

**Authors:** Madhu Priya, Parvendra Singh, Manu Malhotra, Sumeet Angral, Saurabh Varshney, Abhishek Bhardwaj, Amit Kumar Tyagi, Amit Kumar, Manish Kumar Gupta

**Affiliations:** 1 All India Institute of Medical Sciences, Department of Otorhinolaryngology & Head-Neck Surgery, Rishikesh, Uttarakhand, India; 2 All India Institute of Medical Sciences, Department of Paediatric Surgery, Rishikesh, Uttarakhand, India

**Keywords:** Fibrosarcoma, Rhabdomyosarcoma, Soft Tissue Tumor

## Abstract

Soft tissue tumors are not uncommon in childhood and comprise entities that range from common to very rare malignancies. Infantile fibrosarcoma (IFS) is a rare pediatric malignancy mainly seen in the first two years of life. The data about the incidence of infantile fibrosarcoma occurring in the neck in the Indian subcontinent is scarce. To the best of our knowledge, only one case of infant cervical IFS has been reported previously in the Indian subcontinent. We present another case of an eight-year-old male patient with a rapidly growing mass on the left side of the neck. He was successfully treated with a combined modality of surgery and chemotherapy with a good outcome. Among the soft tissue tumors of childhood, IFS is a rare entity. It has a good prognosis and lesser chance of distant metastasis as compared to adult fibrosarcoma. Though surgical excision is the mainstay of treatment, chemotherapy also has a significant role in the treatment of primary tumor and metastasis. We discuss the stated case to bring to the notice this uncommon cause, which can be considered as a differential diagnosis of upper cervical swellings. A better understanding of this entity would help in early diagnosis and aggressive treatment, reducing the overall morbidity and mortality.

## INTRODUCTION

Soft tissue sarcoma accounts for 5-15% of all the malignancies seen in pediatric patients.^1^ Among these sarcomas, 5% are seen in the head and neck region.^1^ The most commonly detected sarcoma in the head and neck region, among children, is the rhabdomyosarcoma.^2^

Among the non-rhabdomyosarcomas, fibrosarcoma is the commonest entity, accounting for about 24.5% of the non-rhabdomyosarcoma seen in Pediatrics. However, fibrosarcoma is more common amongst adults in comparison to children.^3^

In Pediatrics, the presentation of fibrosarcoma is seen in two peaks of age. In the early infancy below the age of one year, and the other peak in children above 5 years of age.^4^ Infantile fibrosarcoma (IFS) is a rare malignant mesenchymal neoplasm of the fibroblasts with an incidence of fewer than 0.2 cases per million in the pediatric age group.^5^ It is a type of non-rhabdomyosarcoma that primarily affects children in the first 2 years of life.^4^ However, it can be present at birth or can develop during early childhood as in our case. There has been an increase in soft tissue sarcoma incidence in Europe but the increase in incidence Is less evident among German population.^6^ It usually manifests as a rapidly growing mass and is commonly seen in the extremities followed by the head and neck, trunk, and, rarely, the pelvis.^7^ ^-^ ^9^

The reported incidence of IFS in the head and neck region is 27% of all cases of IFS.^9^ Although the histological features resemble those of adult fibrosarcoma, the prognosis is better in children.^10^ Surgical resection at the earliest is the treatment of choice. However, some cases of regression on chemotherapy without surgery have been documented.^11^ There is a marked paucity of literature about the incidence of IFS in the neck in the Indian subcontinent. To the best of our knowledge, via Google Scholar and PubMed search using keywords cervical, infantile fibrosarcoma and India, only one case of cervical IFS has been reported previously in the Indian subcontinent, which it was described in a 3-month old baby.^12^ Cervical IFS are more commonly reported In Infants. Our case presented during early childhood.

## CASE REPORT

An 8-year-old child presented with a rapidly progressive swelling in the left upper cervical region for 20 days (Figure 11B). It was mildly painful, with no other systemic complaints. There was no history of any bleeding diathesis. Examination findings were confirmatory of a solitary, mildly tender, globular swelling of approx. 4 x 4 cm at the left level II of the neck. The overlying skin was normal. It was not fluctuant, non-transilluminant. In the oropharynx, a smooth firm bulge was palpable in the left tonsil region, which was bimanually palpable with the neck swelling. Cranial Nerves examination revealed no abnormality. Diagnostic nasal and laryngeal endoscopies were normal. The working diagnosis was a left parapharyngeal space mass of unknown origin.

**Figure 1:**
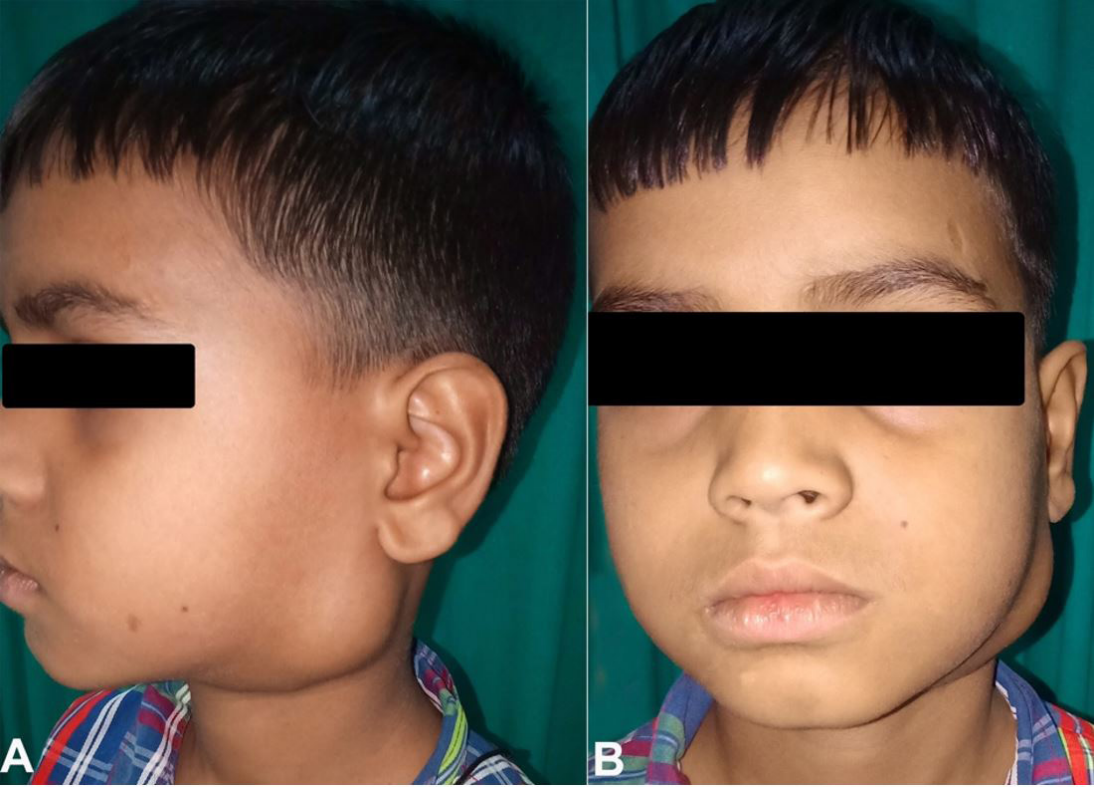
External view of the neck showing swelling over left side of neck In the Infra auricular region (**A –** lateral and **B –** frontal views)

The contrast-enhanced magnetic resonance imaging (CEMRI) revealed a well-defined lobulated mass lesion measuring 4.7 x 4.12 x 6.22 cm in the left superolateral aspect of neck epi-centred at the pre-styloid compartment of the left parapharyngeal space. The lesion was heterogeneously hyperintense to the muscle with a few cystic areas on T2WI and hypointense on T1WI. The lesion abutted the lateral and medial pterygoid muscles, distal external carotid artery with the narrowing of the left internal jugular vein. Posteriorly it displaced the left sternocleidomastoid muscle and the posterior belly of digastric muscle laterally without any infiltration. Inferior extension into the submandibular space without any infiltration was noted. Laterally it displaced the parotid gland (Figure 22B). With these MRI findings, the diagnosis of parapharyngeal space tumor mass was confirmed.

**Figure 2:**
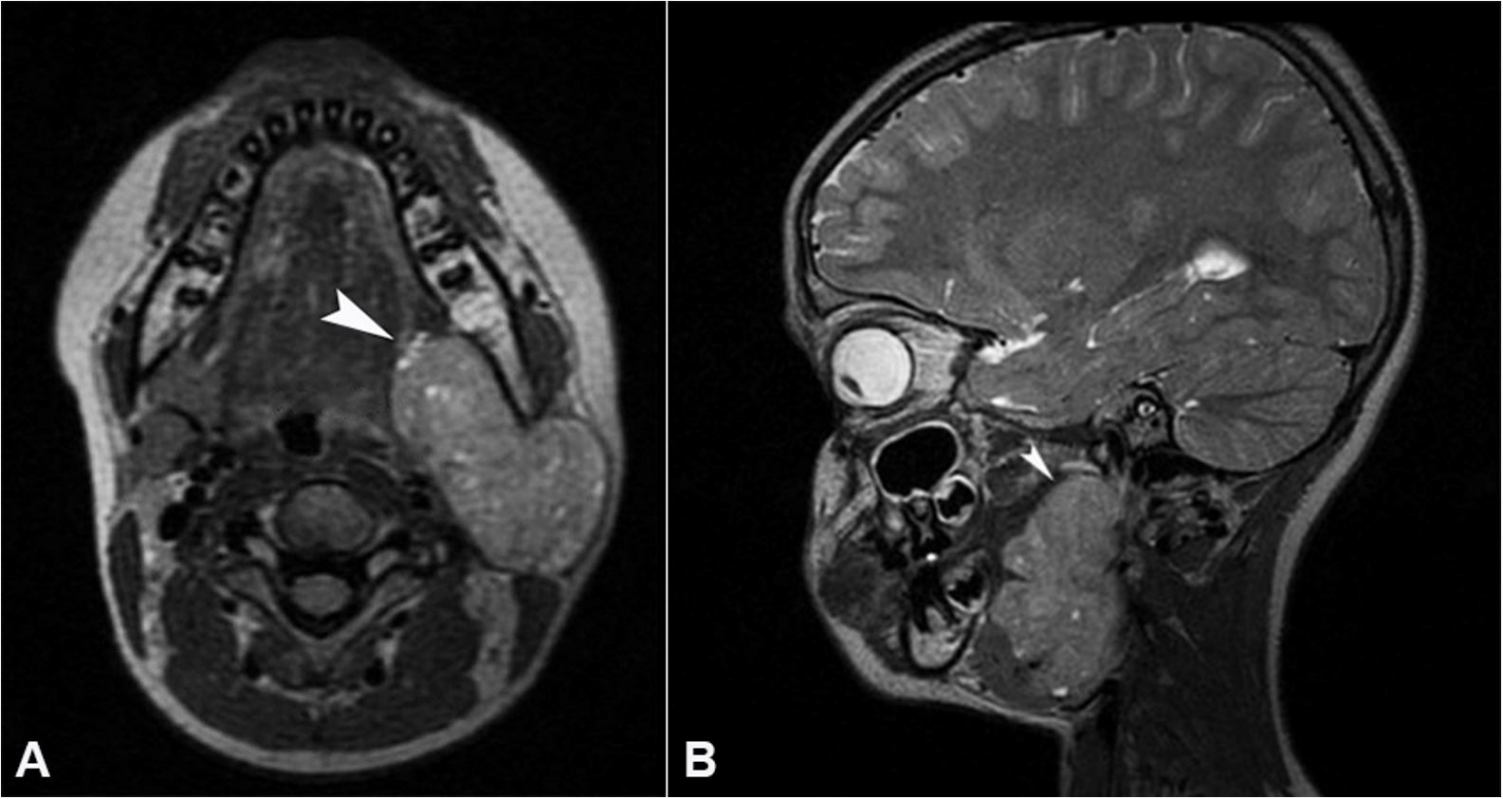
**A and B –** axial and sagittal views respectively showing a well-defined mass in the left side of the neck in the pre-styloid compartment of the left parapharyngeal space.

**Figure 3:**
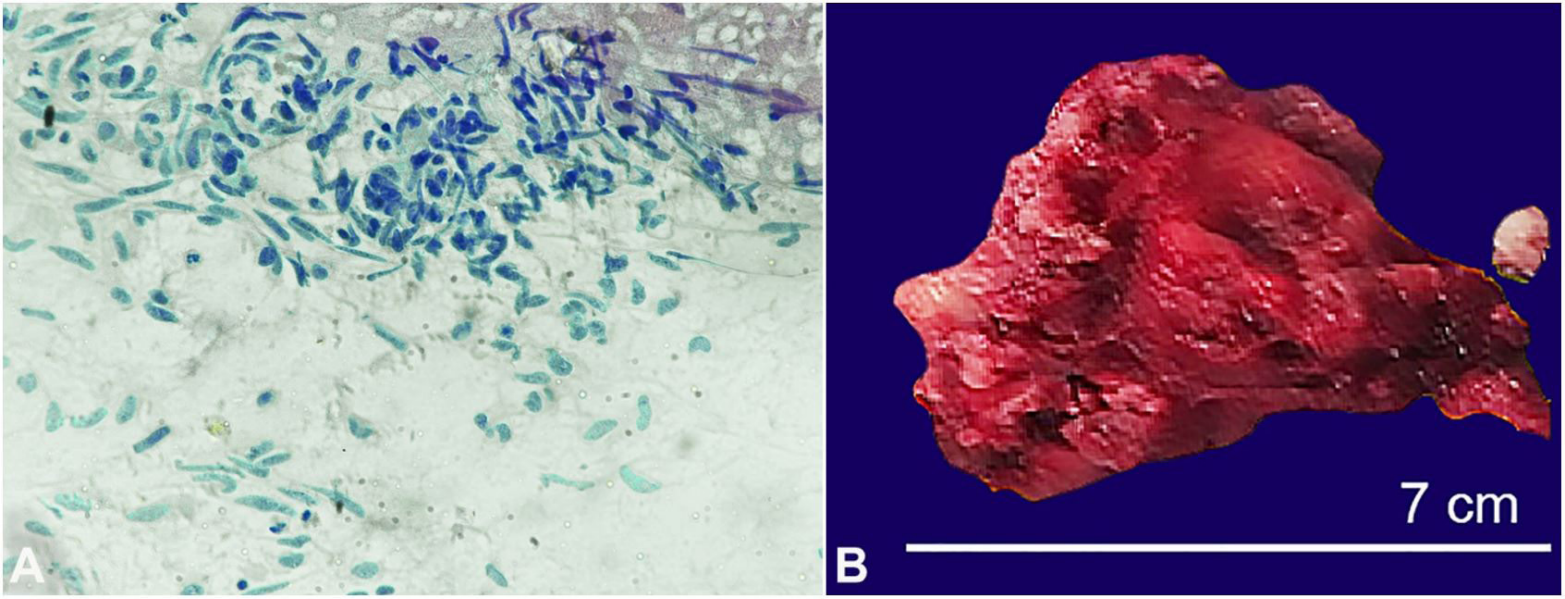
**A –** Photomicrograph of the FNA cytology showing spindle cells in clusters and singly scattered with atypical mitosis in a hemorrhagic background; **B –** Gross specimen of excised tumor showing globular solid mass.

For further evaluation, a fine needle aspiration cytology was done, which showed clusters as well as singly lying bipolar spindle cells with fine, granular chromatin and occasional multinucleated polygonal cells, consistent with a reactive myofibroblastic and fibroblastic proliferation (nodular fasciitis) or spindle cell tumor. (Figure 3A) Based on this cytologic report, a provisional diagnosis of spindle cell tumor was made and an excisional biopsy was advised. To rule out metastases, chest X-ray, abdominal ultrasonography was done, which revealed no signs of metastasis.

The patient underwent surgical excision of the tumor via transcervical approach (Figure 3B).

**Figure 4:**
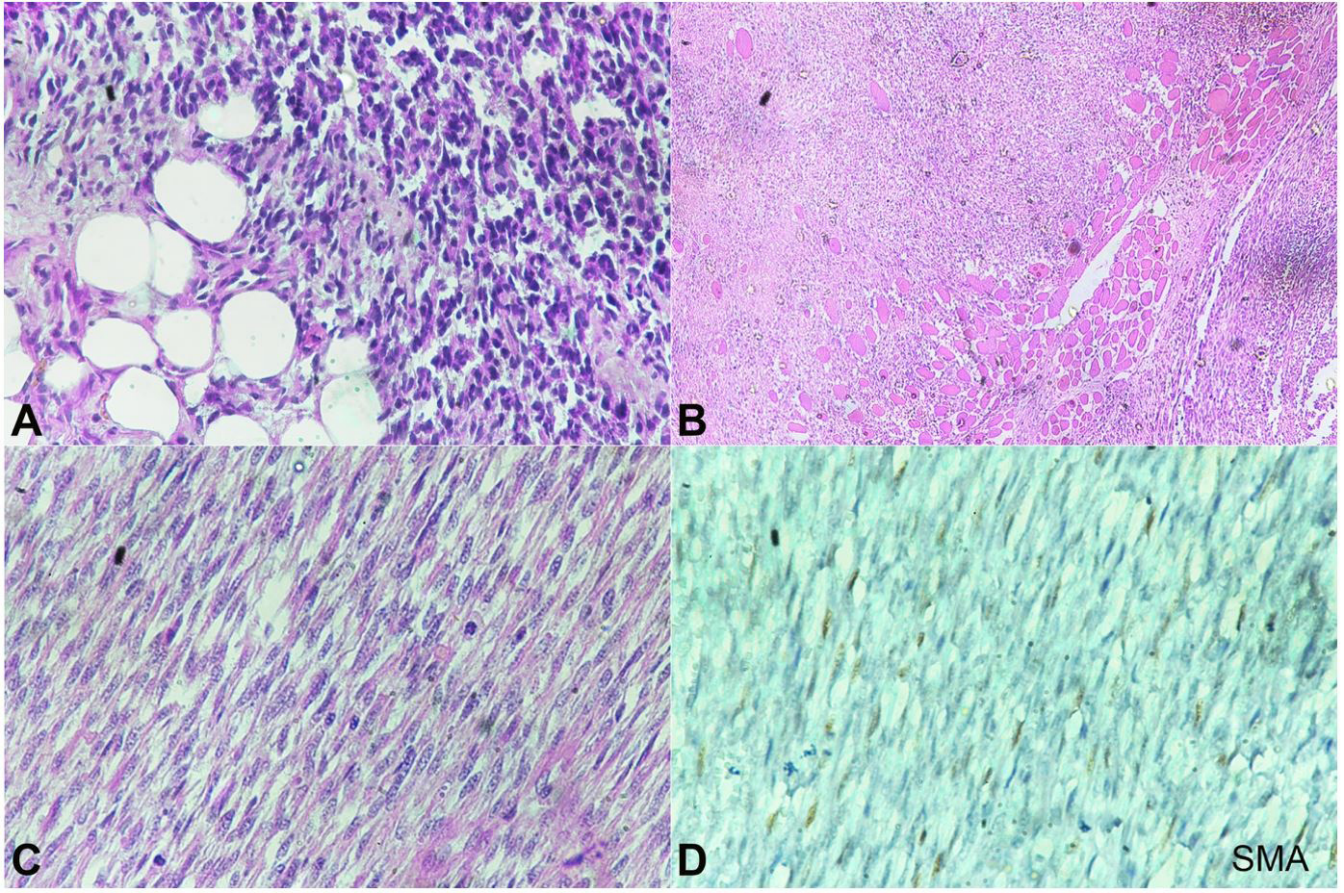
Photomicrographs of the tumor. **A –** Tumor cells invading the subcutaneous fat (H&E, 20X), **B –** muscle infiltration by the tumor (H&E, 20X); **C –** Spindle cell tumor with atypical mitosis (H&E, 40X), **D –** negative reaction to SMA (40X)

**Figure 5:**
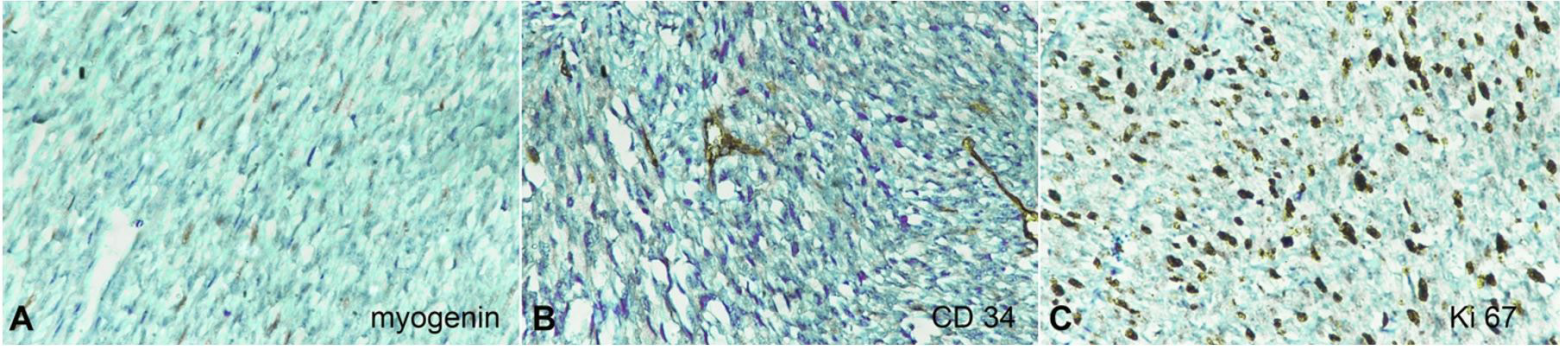
Photomicrographs of the tumor. A – negative reaction for myogenin (40X); **B –** negative reaction for CD 34 (40X); C – Ki67 index of 50-60% (40X).

The surgical specimen measured 6.5 x 5.5 x 5.0 cm, including a surrounding cuff of tissue. The tumor appeared like a globular proliferating growth of 3.0 x 4.0 x 3.0 cm, which was of firm consistency. An intraoperative frozen section revealed fibrocartilaginous tissue with areas of infiltration by spindle and polygonal cells with hyperchromatic nucleus suggesting a poorly differentiated tumor. The tumor was excised with possible widest margins, in view the proximity with vital structures. The postoperative pathological report revealed cellular spindle cell tumor arranged in fascicle, showing herringbone-pattern like appearance. Tumor cells were seen infiltrating the subcutaneous fat and the skeletal muscles. (Figure 44B) The tumor cells were spindled with pleomorphic nuclei showing variable atypia and hyperchromasia. The mitotic figures seen were 8–10 per high power field. The areas of necrosis and hemorrage were identified. (Figure 4C) The superior, inferior, posterior, and medial resected margin were compromised by the tumor. The postoperative histopathology report was confirmatory of spindle cell neoplasm. Tumor cells reacted negative to smooth muscle actin, myogenin and negatively to CD34 (Figures 4 D, 55B). Ki67 index was found to be 50–60% (Figure 5C). Based on these findings, the diagnosis of IFS was made.

The patient was discharged after 7 days of surgery. He was referred to medical oncology for further management, where chemotherapy was planned given the proximity of the tumor to the resected margins.

## DISCUSSION

Tumors of soft tissue are commonly seen in childhood,^5^ ^,^ ^6^ which are mostly benign, with a good cure rate followed by a surgical excision. Malignant mesenchymal neoplasms are rare tumors in childhood, accounting to less than 1% of the overall malignant tumors in humans.^13^ These malignant tumors are life-threatening and may pose a diagnostic and therapeutic challenge because of the varied spectrum in clinical features and histopathology. More than 50 types of the smooth tissue tumor have been identified so far. These are often associated with unique clinical, prognostic, and therapeutic features. Due to recent advances in various diagnostic modalities like immunohistochemistry, the understanding of these tumors has significantly improved, both from a histopathological and a genetic point of view. The close association of pathologists, surgeons and oncologists has brought about a significant improvement in the treatment and survival of patients, with the overall 5-year survival rate rising from almost fatal to 65-75%.^13^

Sarcomas are spindle cell malignancies of mesenchymal cell origin and are named and classified after the predominant cell line that is present. For example, in the bone (osteosarcoma), cartilage (chondrosarcoma), smooth muscle (leiomyosarcoma), skeletal muscle (rhabdomyosarcoma) and fibroblast (fibrosarcoma). These tumors vary considerably in presentation, treatment, and prognosis. At the same time, there are characteristics shared by many sarcomas. Since all are connective tissue in origin, they are called soft tissue tumors.

Conventional fibrosarcoma show bimodal age distribution with adult and Infantile types. Fibrosarcoma is an intermediate-grade tumor. Though, histopathologically similar, IFS exhibit a predominantly benign course in sharp contrast to its adult counterpart. The reported incidence of IFS is less than 0.2 cases per million in the pediatric age group.^5^ IFS is a rare form of pediatric soft tissue sarcoma. IFS occurring in the head and neck region amounts to 27% of all cases of IFS.^9^ Though malignant, at times, it exhibits a benign clinical course. It is a rapidly growing tumor which progresses in the deep soft tissues of the extremities or trunk. Though rare, it can present with distant metastases and lymph node involvement.^14^ ^-^ ^16^ Among all the cases of infantile fibrosarcoma, almost 60% of the cases are diagnosed in infancy before the age of 3 months. About 30-50% of these tumors are present at birth.^5^

The histologic features of these lesions do not always correlate well with clinical behavior. Histopathologically, it is characterized by densely arranged cellular spindles with a high proliferative index with areas of necrosis arranged in an intersecting bundle with a herringbone pattern. The tumor cells have a variable fibroblastic and myofibroblastic ultrastructure and a nonspecific immunophenotype with substantial positivity for Desmin and actin. IFS is characterized by the recurrent translocation t (12;15) (p13; q25) with the transcript ETV6-NTRK3^17^ ^,^ ^18^, and a significant receptor tyrosine kinase activation (PI3-Akt, MAPK, and SRC activation) has been reported.^19^

Clinical presentation of infantile fibrosarcoma is usually a rapidly progressing mass involving trunk or extremities, which can often give a grotesque appearance to the child. These tumors are rare in the head and neck and often present as neck swelling, which significantly increases in size in a few days. The swelling can cause compressive symptoms due to the mass effect. Though rare, these tumors can present as metastatic disease. For diagnosis, various imaging modalities are available, which are chosen according to the involved site. Fine needle aspiration cytology can be performed as a preoperative workup. For ruling out metastasis chest radiograph, abdominal ultrasound, computed tomography, and bone scan can be performed.

Early diagnosis of IFS in the pediatric population can reduce morbidity and mortality. Surgical resection of the primary tumor is the mainstay of treatment. The current management of IFS has evolved to include initial biopsy and chemotherapy with delayed conservative resection planned after tumor shrinkage.^20^ Occasionally, tumors may undergo spontaneous regression with chemotherapy alone, sparing patient from surgery. In the cases submitted firstly to surgical resection, an adjuvant chemotherapy may not be required. The European soft tissue sarcoma group guidelines recommended close surveillance for patients undergoing upfront surgery with negative histopathological margins.^21^ The role of radiotherapy is of limited value as the bone growth in limbs would be severely compromised. ETV6-NTRK3 transcript may represent a potential therapeutic target but is not specific to IFS and has been identified in other tumors, including secretory breast carcinoma and mammary analogue secretory carcinoma, acute lymphoblastic leukemia, and high-grade gliomas.^22^ Recurrence of IFS is common, but the rates of metastasis are less than 10% in children younger than 5 years of age and 50% in children more than 10 years of age.^23^

## CONCLUSION

IFS is a rare tumor amongst the pediatric population and head & neck is an uncommon site of its presentation. The disease is life-threatening. It may pose a diagnostic and therapeutic challenge because of the varied spectrum of clinical features and histopathology. Immunohistochemistry and histopathology are the main aids to diagnosis apart from the clinical behavior of the pathology. It should be kept as a differential diagnosis as prognosis is better compared to its adult counterpart.
